# Knockdown of *Foxg1* in Sox9+ supporting cells increases the trans-differentiation of supporting cells into hair cells in the neonatal mouse utricle

**DOI:** 10.18632/aging.104009

**Published:** 2020-10-24

**Authors:** Yuan Zhang, Shasha Zhang, Zhonghong Zhang, Ying Dong, Xiangyu Ma, Ruiying Qiang, Yin Chen, Xia Gao, Chunjie Zhao, Fangyi Chen, Shuangba He, Renjie Chai

**Affiliations:** 1 MOE Key Laboratory for Developmental Genes and Human Disease, School of Life Sciences and Technology, Jiangsu Province High-Tech Key Laboratory for Bio-Medical Research, Southeast University, Nanjing, China; 2 Co-Innovation Center of Neuroregeneration, Nantong University, Nantong, China; 3 Institute for Stem Cell and Regeneration, Chinese Academy of Science, Beijing, China; 4 Beijing Key Laboratory of Neural Regeneration and Repair, Capital Medical University, Beijing, China; 5 Department of Ophthalmology, Zhongda Hospital, Southeast University, Nanjing, China; 6 Department of Otolaryngology Head and Neck Surgery, Affiliated Drum Tower Hospital of Nanjing University Medical School, Jiangsu Provincial Key Medical Discipline (Laboratory), Nanjing, China; 7 Department of Biomedical Engineering, Southern University of Science and Technology, Shenzhen, China; 8 Department of Otolaryngology Head and Neck, Nanjing Tongren Hospital, School of Medicine, Southeast University, China

**Keywords:** Foxg1, Sox9, supporting cells, hair cell regeneration, trans-differentiation

## Abstract

Foxg1 plays important roles in regeneration of hair cell (HC) in the cochlea of neonatal mouse. Here, we used Sox9-CreER to knock down *Foxg1* in supporting cells (SCs) in the utricle in order to investigate the role of Foxg1 in HC regeneration in the utricle. We found Sox9 an ideal marker of utricle SCs and bred Sox9^CreER/+^Foxg1^loxp/loxp^ mice to conditionally knock down *Foxg1* in utricular SCs. Conditional knockdown (cKD) of *Foxg1* in SCs at postnatal day one (P01) led to increased number of HCs at P08. These regenerated HCs had normal characteristics, and could survive to at least P30. Lineage tracing showed that a significant portion of newly regenerated HCs originated from SCs in *Foxg1* cKD mice compared to the mice subjected to the same treatment, which suggested SCs trans-differentiate into HCs in the *Foxg1* cKD mouse utricle. After neomycin treatment *in vitro*, more HCs were observed in *Foxg1* cKD mice utricle compared to the control group. Together, these results suggest that *Foxg1* cKD in utricular SCs may promote HC regeneration by inducing trans-differentiation of SCs. This research therefore provides theoretical basis for the effects of Foxg1 in trans-differentiation of SCs and regeneration of HCs in the mouse utricle.

## INTRODUCTION

The mammalian utricle, one of the vestibular sensory organs, uses hair cells (HCs) to detect linear acceleration such as gravity and body movement [1, 2]. The utricle contains two types of HCs (type I and type II), which have distinct morphologies, ion channels, and synaptic features [3]. Type I HCs are predominantly found in the J-shaped striolar (S) region (in the central part of the utricle), and type II HCs are mainly found in the extrastriolar (ES) region (in the peripheral part of the utricle) [4, 5]. Both type I and type II HCs can be replenished to restore vestibular function after injury in the avian utricle, while mammalian vestibular HC regeneration does not occur spontaneously but can be induced to occur through different approaches [6–11].

Another main cell type in the utricle is supporting cells (SCs), which have the potential to proliferate and regenerate new HCs in the injured mammalian utricle [12–14], and some populations of SCs express HC-specific transcription factors after injury to HCs in both the cochlea and the utricle [8–11, 15, 16]. In contrast to the very limited regenerative capacity in mammals, non-mammalian SCs possess a natural regenerative capacity through two distinct mechanisms [17, 18]. One is mitotic regeneration, in which SCs re-enter the cell cycle, proliferate, and differentiate into new HCs. The other is direct trans-differentiation, in which the surrounding SCs switch their cell fates to directly differentiate into HCs [19–21]. However, the genes and pathways involved in HC regeneration are not yet clearly defined. Therefore, it is necessary to identify the genes and pathways that direct HC regeneration in order to identify new therapeutic targets for preventing and treating vestibular dysfunction.

Foxg1 is a vital transcription factor that is expressed in many kinds of neural and sensory tissues, such as the cerebral cortex, telencephalon, eyes, and inner ear [22–27]. In the forebrain, Foxg1 maintains the pools of progenitor cells and can inhibit the differentiation ability of neurons, and Foxg1 expression is decreased when the neuronal progenitors undergo neuronal differentiation [28, 29]. In addition, Foxg1 deficiency leads to gliogenesis and neurogenesis in postnatal mice [30]. In the telencephalon, Foxg1 contributes to the promotion of neural precursor proliferation, and mutations in *Foxg1* lead to decreased proliferation and premature differentiation of telencephalic progenitors [31, 32]. In the eyes, Foxg1 plays important roles in optic fissure closure, ciliary margin tissue formation, and retinal ganglion cell projection [26, 33–35]. In the auditory organ, Foxg1 is widely expressed in the utricle, cochlea, and saccule and is essential for the morphology and histology of the inner ear [36, 37]. Importantly, Foxg1 staining in the utricle shows that it is expressed in some neuronal HCs and in all SCs [37]. Our recent studies showed that *Foxg1* cKD in SCs induces the HC regeneration in neonatal mice by promoting the trans-differentiation of SCs in the cochlea [38].

Based on the roles of Foxg1 described above, we make a hypothesis that Foxg1 also regulates the differentiation of SCs into HCs in the neonatal mice utricle. Sox9 (sex-determining region Y-box 9), Sox2 (sex-determining region Y-box 2), Lgr5 (leucine-rich repeat-containing G protein-coupled receptor 5), and Plp (proteolipid protein 1) have been reported to be expressed in utricular SCs [17, 39, 40]. Sox2 is reported to be expressed in almost all SCs in the utricle [41, 42], but it is also highly expressed in type II HCs in the utricle [41, 42] and thus is not a good marker for SCs in the utricle. Lgr5 is only expressed in the SCs of the S region in the neomycin-damaged utricle [17], and Plp is mainly expressed in ES SCs in the utricle and its expression level declines with age [43]. Among all these markers, only Sox9 is expressed in all SCs and is barely expressed in HCs in the utricle [39], and thus we mainly used Sox9 to study the effect of Foxg1 in SCs in the utricle. We crossed Sox9^CreER/+^ mice with Foxg1^loxp/loxp^ mice and injected tamoxifen to specifically knock down *Foxg1* in Sox9+ cells in the utricle of neonatal mice. By evaluating the differentiation ability of the *Foxg1* cKD SCs, the results showed that *Foxg1* cKD in Sox9+ SCs promoted the direct trans-differentiation ability of SCs under either normal or neomycin-damaged conditions. Our study describes the effects of Foxg1 in HC regeneration in the utricle of mice and suggests that Foxg1 may be a therapeutic target for preventing vestibular dysfunction.

## RESULTS

### *Foxg1* is conditionally knocked down in utricular SCs in Sox9-CreER mice

**Figure 1:**
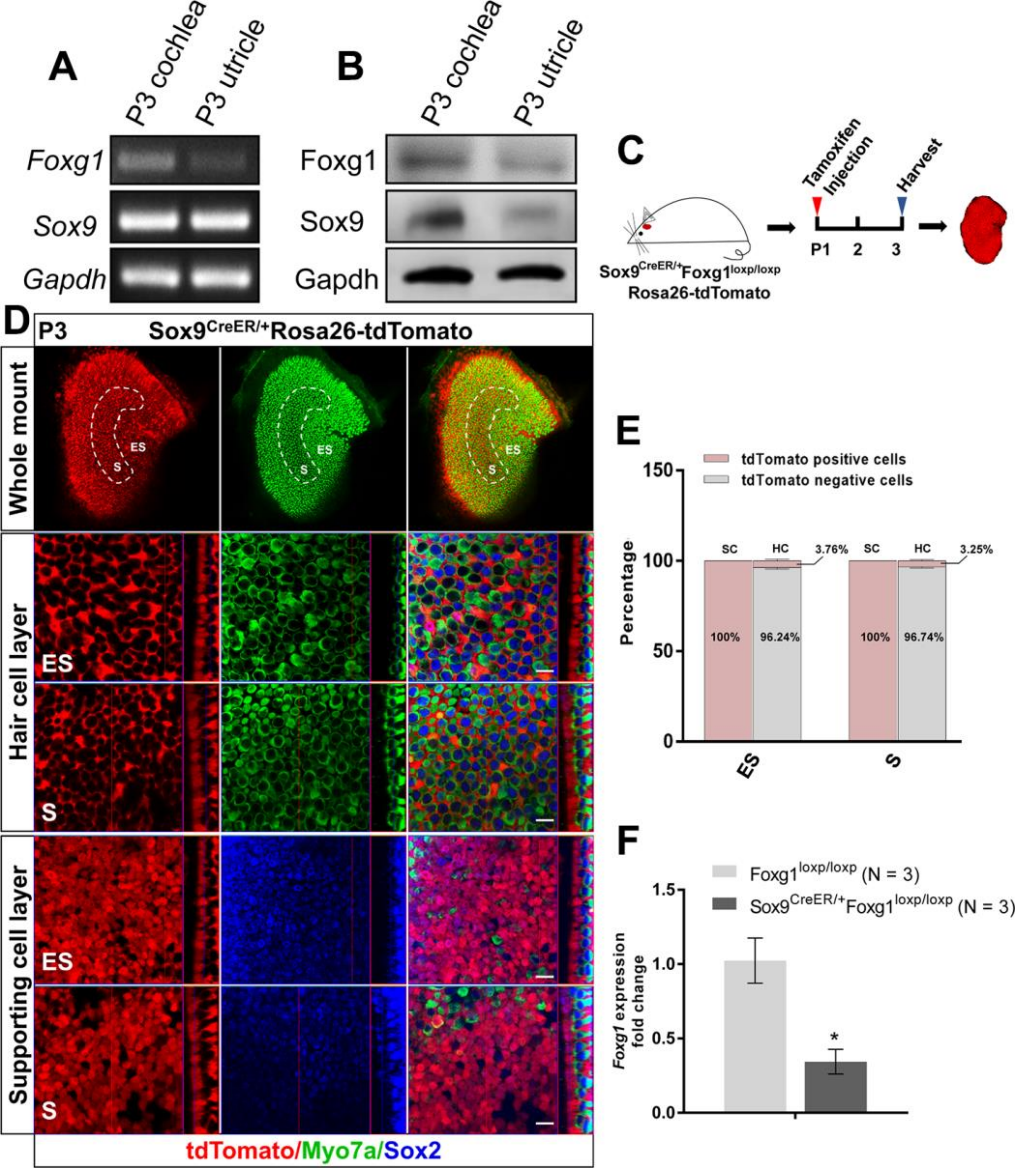
**cKD of *Foxg1* in Sox9+ SCs in the mouse utricle.** (**A**, **B**) *Sox9* and *Foxg1* mRNA (**A**) and protein (**B**) expression in P3 mouse cochlea and utricles as detected by RT-PCR and Western blotting, respectively. (**C**) P01 mice were i.p. injected with tamoxifen and the utricle was harvested at P3 to observe the Sox9 expression pattern in the utricle. (**D**) Lineage tracing showed Sox9 expression mainly in the SCs of the P3 mouse utricle. Myo7a (green) was used to indicate the HC, and Sox2 (blue) was used to label SCs. Scale bar, 10 μm. (**E**) Quantification of the percentage of tdTomato positive and negative cells in both the HC layer and SC layer of the ES and S regions of the utricle. (**F**) *Foxg1* cKD efficiency was measured by qPCR. mRNA was extracted from the whole utricle. N indicates the number of real-time qPCR experimental repetitions. *p < 0.05, data are represented as mean ± SEM.

Foxg1 is widely expressed in the inner ear, including the utricle [37]. Therefore, we first confirmed the expression of Foxg1 in the utricle by RT-PCR and western blotting (Figure 1A and 1B). Because we previously reported that cKD of *Foxg1* in cochlear SCs using Sox2-CreER mice induces increased HC numbers in neonatal mice [38], we hypothesized that Foxg1 has a similar function in the utricle as it does in the cochlea. However, although Sox2 is expressed in almost all of the SCs in the utricle, it is also expressed in some of the type II HCs in the utricle [41, 42], and thus Sox2-CreER mice could not be used to conditionally knock down *Foxg1* in the SCs in the utricle. We therefore tested two transgenic CreER mice, Sox9-CreER and Plp-CreER mice, to determine which would be more suitable for our study. By injecting tamoxifen into Plp^CreER/+^Rosa26-tdTomato mice, we found that Plp was mostly expressed in SCs of the ES region and only slightly expressed in SCs of the S region (Supplementary Figure 2), similar to what was reported previously [43]. We also injected tamoxifen into Sox9^CreER/+^Rosa26-tdtomato mice and found that Sox9 was expressed in almost all of the SCs in the utricle (Figure 1C and 1D) but in only 3.76% and 3.25% of the HCs in the ES and S regions, respectively (Figure 1E), which meant that Sox9 could be considered a marker of utricular SCs and that we could use Sox9-CreER mice to conditionally knock down *Foxg1* in utricular SCs. Sox9-CreER mice and Foxg1-loxp mice were crossed to obtain Sox9^CreER/+^Foxg1^loxp/loxp^ mice and conditionally knocked down *Foxg1* by injecting tamoxifen at P01. Our qPCR results showed that *Foxg1* was successfully knocked down by 66% in the utricle (Figure 1F).

### *Foxg1* cKD in SCs increases the number of HCs in the neonatal mouse utricle

**Figure 2:**
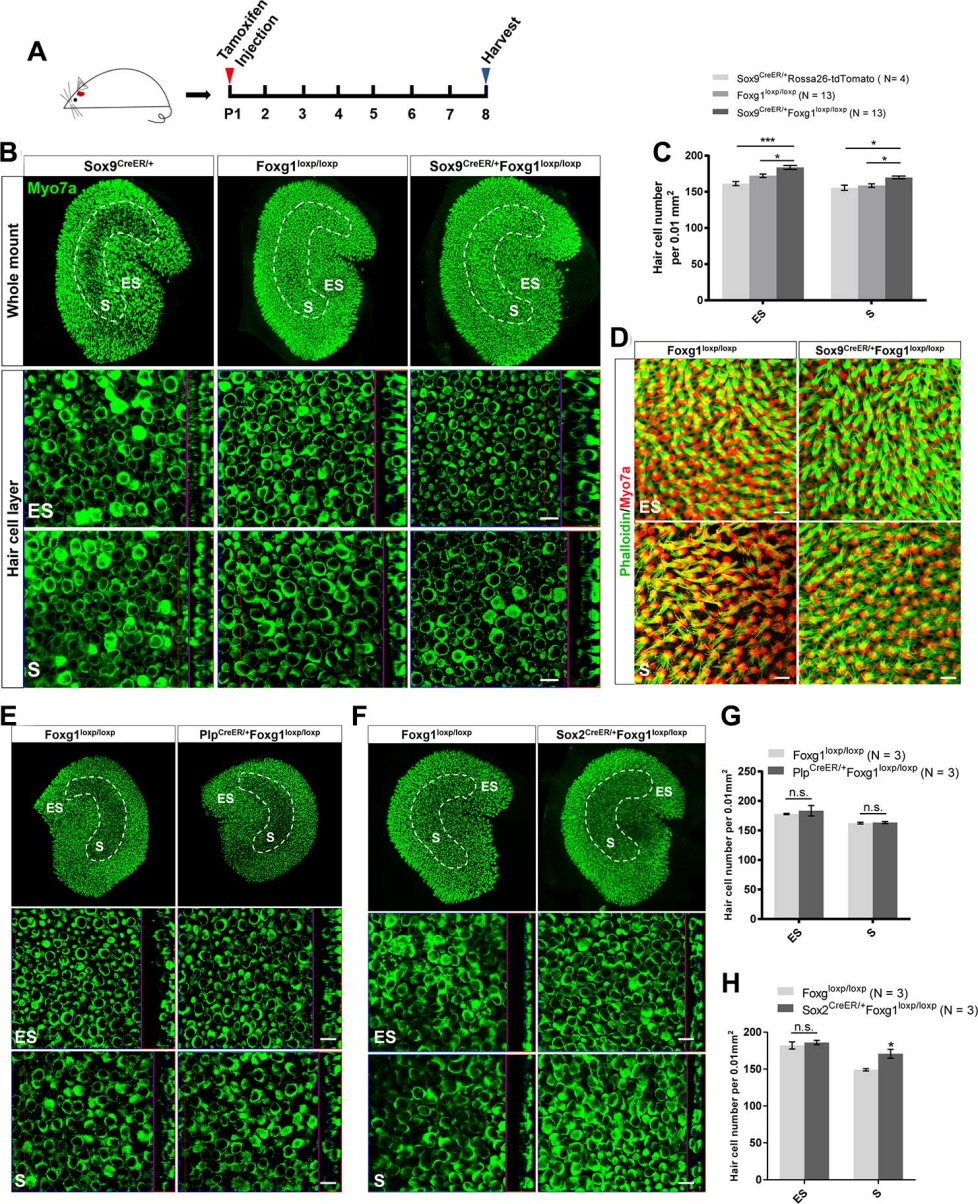
**cKD of *Foxg1* in Sox9+ SCs led to increased HCs number in the mouse utricle at P08.** (**A**) Tamoxifen was injected into P01 mice to knock down *Foxg1* in Sox9+ SCs, and the utricle was harvested at P08. (**B**) Immunofluorescence staining with anti-Myo7a (green) antibodies in the utricle of P08 Sox9^CreER/+^, Foxg1^loxp/loxp^, and Sox9^CreER/+^Foxg1^loxp/loxp^ cKD mice. (**C**) Quantification of HCs in the ES and S regions of the utricle. *p < 0.05, ***p < 0.001. (**D**) Immunofluorescence staining with phalloidin (green) in the utricle of P08 *Foxg1* cKD and control mice. (**E**, **F**) Immunofluorescence staining with anti-Myo7a (green) antibodies in the utricle from P8 Plp^CreER/+^Foxg1loxp/loxp (**E**) and Sox2^CreER/+^Foxg1^loxp/loxp^ mice (**F**). Foxg1^loxp/loxp^ mice were used as the control mice. (**G**, **H**) Quantification of the total numbers of HCs in the ES and S regions of the utricle from the above mice. *p < 0.05, n.s. not significant. For all experiments, Myo7a was used to indicate the HC. Scale bar, 10 μm. N indicates the number of mice, data are represented as mean ± SEM.

As previously reported, *Foxg1* cKD in cochlear SCs leads to increased number of HCs [38]. Here we injected tamoxifen into P01 Sox9^CreER/+^Foxg1^loxp/loxp^ mice to specifically knock down *Foxg1* in utricular SCs and we harvested utricle at P08 to observe their phenotype (Figure 2A). Compared to the Sox9^CreER/+^ and Foxg1^loxp/loxp^ control mice, we observed significantly more HCs in both the S and ES regions of the Sox9^CreER/+^Foxg1^loxp/loxp^ mouse utricle (Figure 2B and 2C). In addition, we also used phalloidin to observe the hair bundles of utricular HCs and found that the hair bundles of Foxg1 cKD mice were morphologically normal at P08 (Figure 2D). Moreover, cKD *Foxg1* in Plp+ SCs did not significantly change the number of hair cells both in S and ES region of utricle. However, we observed increased HC numbers in the S region of utricle when cKD *Foxg1* in Sox2+ SCs (Figure 2E–2H) at P08. All of these results were consistent with the increase in HC number in the *Foxg1* cKD cochlea, thus suggesting that Foxg1 plays similar roles in both cochlear and utricular SCs and HC regeneration, and thus cKD of *Foxg1* probably also increases the HC number in the utricle by inducing direct trans-differentiation of SCs as it does in the cochlea.

### cKD of *Foxg1* in utricular SCs induces the direct trans-differentiation of SCs into HCs

**Figure 3:**
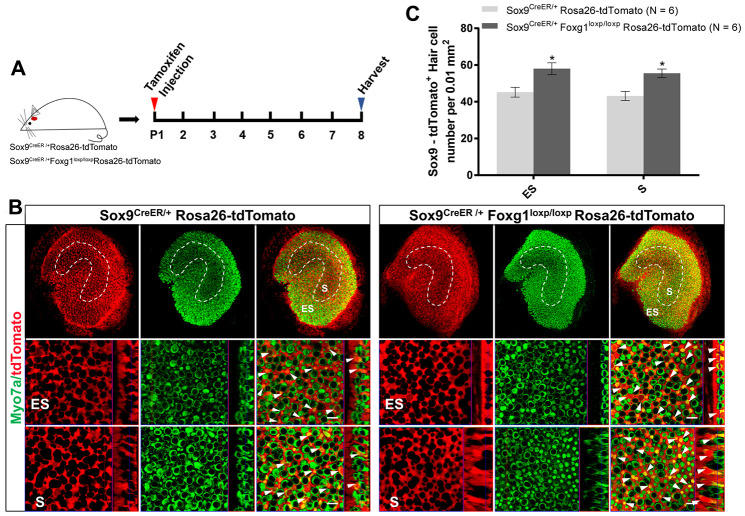
***Foxg1* cKD led to increased trans-differentiation of Sox9+ SCs in the utricle *in vivo*.** (**A**) P01 mice were i.p. injected Tamoxifen for activating the Cre enzyme, and the mice were sacrificed at P08. (**B**) Immunofluorescence staining with anti-Myo7a (green) antibodies in the utricle from P08 Sox9^CreER/+^Rosa26-tdTomato and Sox9^CreER/+^Foxg1^loxp/loxp^Rosa26-tdTomato mice. Myo7a was used as the HC marker. tdTomato+ HCs are indicated by white arrows. (**C**) Quantification of tdTomato+ HCs per 0.01 mm^2^ area in both the S and ES regions of the P08 mouse utricle. Scale bar, 10 μm. N indicates the number of mice. *p < 0.05, data are represented as mean ± SEM.

To test our hypothesis, we crossed Sox9^CreER/+^Foxg1^loxp/loxp^ mice with Rosa26-tdTomato mice to get Sox9^CreER/+^Foxg1^loxp/loxp^Rosa26-tdTomato mice and injected tamoxifen at P01 to lineage trace the utricular SCs (Figure 3A). We observed significantly more newly generated tdTomato+ HCs in Sox9^CreER/+^Foxg1^loxp/loxp^Rosa26-tdTomato mice compared to Sox9^CreER/+^Rosa26-tdTomato control mice (Figure 3B and 3C). These results are also consistent with the increased tdTomato+ HC number in the *Foxg1* cKD cochlea, thus suggesting that cKD of *Foxg1* also induces direct trans-differentiation of utricular SCs into HCs.

### *Foxg1* cKD in SCs increases the number of utricular HCs at least to P30

**Figure 4:**
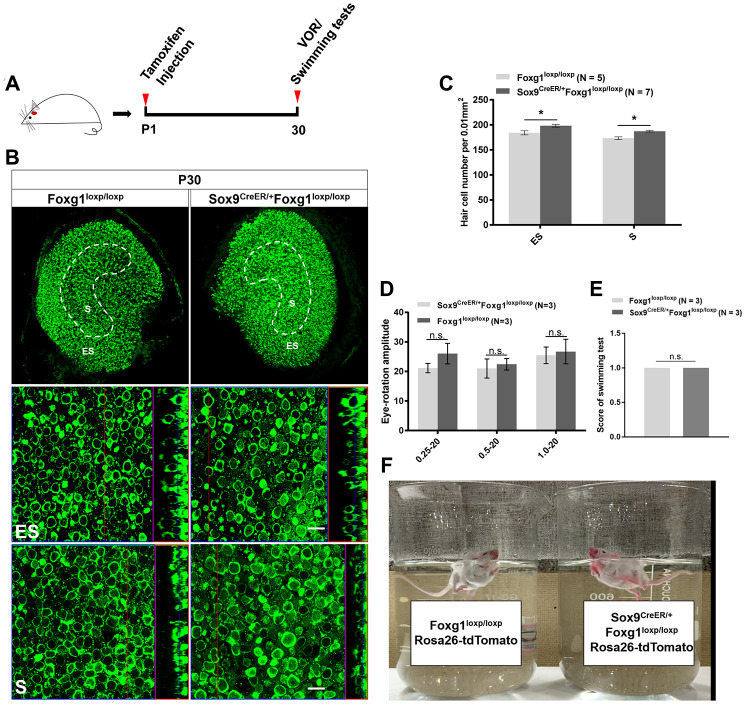
**P30 *Foxg1* cKD mice showed increased numbers of utricular HCs and normal VOR response and swimming behavior.** (**A**) P01 mice were i.p. injected Tamoxifen, and at P30 the mice were subjected to VOR and swimming tests and then sacrificed for immunofluorescence staining. (**B**) Immunofluorescence staining with anti-Myo7a (green) antibodies in the utricle from P30 Foxg1^loxp/loxp^ and Sox9^CreER/+^Foxg1^loxp/loxp^ cKD mice. (**C**) Quantification of the HCs number in both the ES and S regions of the P30 mouse utricle. (**D**) Mice were positioned in the VOR testing system, and their VOR responses were measured. There was no significant difference in eye rotation amplitude between Foxg1^loxp/loxp^ and Sox9^CreER/+^Foxg1^loxp/loxp^ cKD mice at three stimulation frequencies (0.25 Hz, 0.5 Hz, and 1.0 Hz). (**E**) Score of swimming test of Foxg1^loxp/loxp^ and Sox9^CreER/+^Foxg1^loxp/loxp^ at P30. *Foxg1* cKD mice showed the same score compared to the control mice. (**F**) A single frame from the video of the swimming tests of Foxg1loxp/loxpRosa26-tdTomato and Sox9CreER/+Foxg1loxp/loxpRosa26-tdTomato mice at P30. Both *Foxg1* cKD mice and the control mice showed normal swimming behavior. For all experiments, “N” indicates the number of mice. Scale bar, 10 μm. *p < 0.05, n.s. no significance, data are represented as mean ± SEM.

Considering that extra HCs can survive to at least P30 in the cochlea, we also studied the survival of newly generated HCs in the utricle. After injecting tamoxifen into Sox9^CreER/+^Foxg1^loxp/loxp^ mice at P01, there were still significantly more HCs at P30 in both the S and ES regions compared to the control mice (Figure 4A–4C). Because the utricle is required for detecting linear acceleration, we used VOR and swimming tests to study the sense of balance in *Foxg1* cKD mice (Figure 4D–4F and Supplementary Video 1). We did not detect any eye-rotation amplitude differences or abnormal swimming behaviors between *Foxg1* cKD mice and control mice, which means that the increased number of HCs in the utricle did not affect the mice’s sense of balance.

### In an *in vitro* model of neomycin damage, cKD of *Foxg1* also increased the number of HCs by inducing the trans-differentiation of SCs

**Figure 5:**
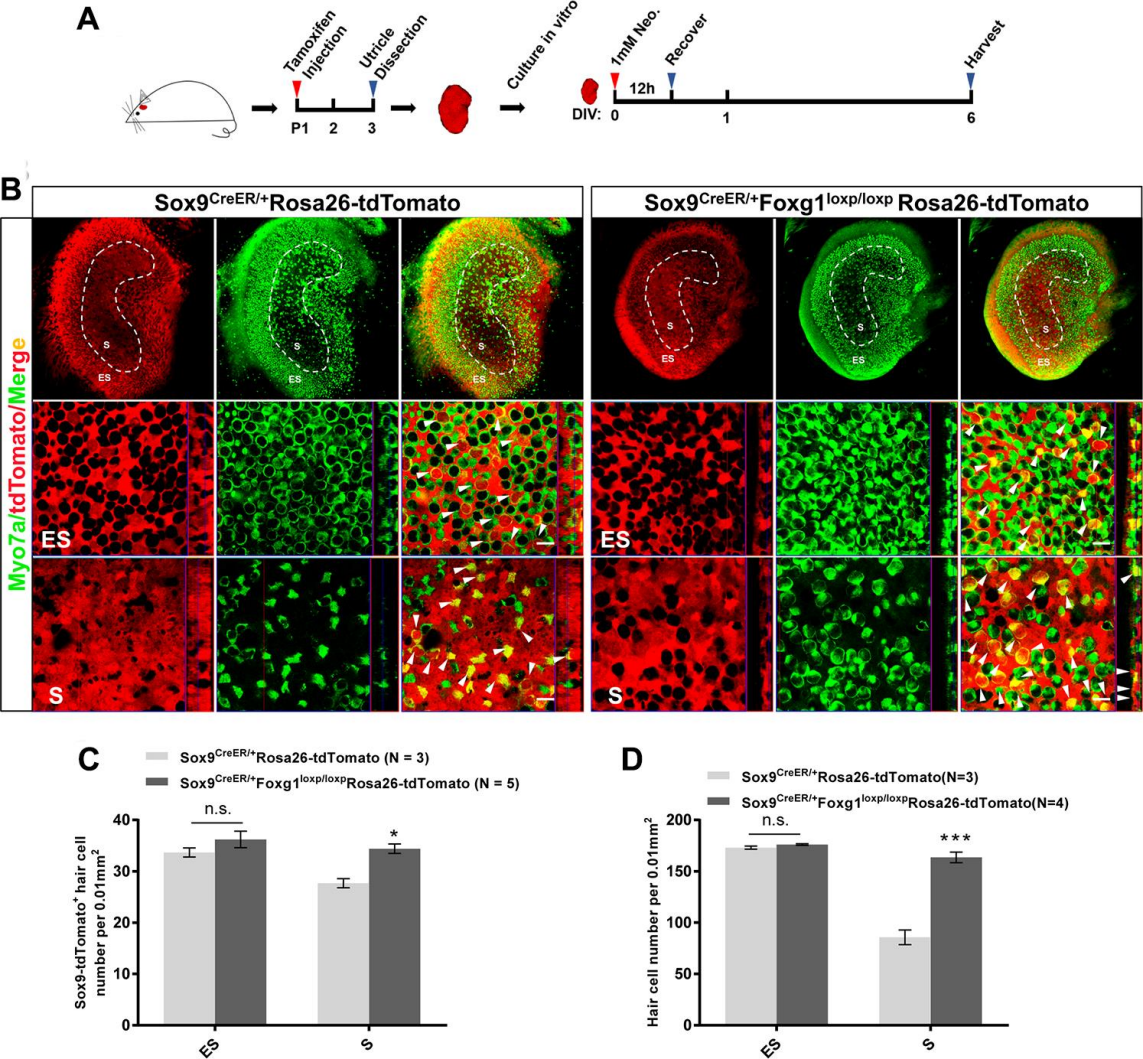
***Foxg1* cKD induced the trans-differentiation of Sox9^+^ SCs in the mouse utricle after neomycin injury *in vitro*.** (**A**) P01 mice were i.p. injected Tamoxifen to activate the Cre enzyme, and at P3 the utricle were harvested and cultured *in vitro*. Neomycin (1 mM) was added to the culture medium for 12 h, and the utricle was allowed to recover for 5 days and then harvested at day 6 *in vitro*. DIV, days in vitro. (**B**) Immunofluorescence staining with anti-Myo7a (green) antibodies in the cultured utricle from Sox9^CreER/+^Rosa26-tdTomato and Sox9^CreER/+^Foxg1^loxp/loxp^Rosa26-tdTomato mice. Myo7a was used as the HC marker. tdTomato+ HCs are indicated by white arrows. (**C**) Quantification of tdTomato+ HCs in the S and ES regions per 0.01 mm^2^ area of the utricle. (**D**) Quantification of the total number of HCs in the S and ES regions per 0.01 mm^2^ area of the utricle. Myo7a was used to indicate the HCs. Scale bar, 10 μm. “N” indicates the number of mice. *p < 0.05, ***p < 0.001; n.s., no significance, data are represented as mean ± SEM.

Neomycin treatment leads to HC loss in the utricle, especially in the S region, and there is very limited HC regeneration after injury in the utricle [11, 17, 44]. In order to study the HC regeneration ability of *Foxg1* cKD SCs in the utricle, we injected tamoxifen into Sox9^CreER/+^Foxg1^loxp/loxp^Rosa26-tdTomato mice, dissected and cultured their utricles *in vitro*, and lineage traced the SCs after neomycin-induced HC damage (Figure 5A). The Sox9^CreER/+^Rosa26-tdTomato mice were used as control mice, which were also injected with tamoxifen. Their utricles were also dissected, cultured *in vitro* and treated with neomycin. After 6 days of culture, we found significantly more HCs and tdTomato+ HCs in the S region of Sox9^CreER/+^Foxg1^loxp/loxp^Rosa26-tdTomato mice compared to control mice (Figure 5B–5D), which was. consistent with the *in vivo* data showing increased numbers of HCs and SC trans-differentiation in *Foxg1* cKD utricles. But we did not found any changes in HC or tdTomato+ HCs in the ES region (Figure 5B–5D), which may due to more serious damage in the S region than the ES region in the utricle [17, 45]

## DISCUSSION

Foxg1 is involved in the development of various organs such as the forebrain, cerebral cortex, etc. Lack of Foxg1 in the forebrain leads to defects in ventral telencephalon and dorsal structures due to the abnormal proliferation and differentiation of neuroepithelial cells [22, 28, 29, 46–49]. In addition, Foxg1 also contributes to the maintenance of hippocampal dentate gyrus progenitor cells and to the process of neurogenesis and glialogenesis [30]. Foxg1 not only play roles in CNS organs, but also in retina, otic vesicles and olfactory placodes [50–52]. Foxg1 also contributes to the development of the inner ear and can induce HC regeneration through promoting direct trans-differentiation of SCs in the cochlea, and *Foxg1* knockout mice show shortened cochlea with increased rows of HCs and abnormal vestibular innervation [37, 38, 53]. Our previous study reported that cKD of *Foxg1* in SCs resulted in an increase in HC number by inducing trans-differentiation of SCs in the cochlea [38]. However, the effects of Foxg1 in trans-differentiation of SCs in the utricle remain unclear. Based on these previous studies, we hypothesized that Foxg1 also plays important roles in the utricle of the vestibular system.

The sense of balance requires mechanosensory HCs, and vestibular HC degeneration (due to genetic mutations, ototoxic drugs, etc.) is the most important cause of balance disorders [54]. The development, function, and maintenance of sensory HCs is heavily dependent on the non-sensory SCs that surround the HCs [55], and it has been shown that HCs in the utricle are regenerated mostly from SCs [17, 56, 57]. Therefore, understanding the detailed mechanisms about HC regeneration through regulating SCs will be very important for the treatment of vestibular disorders caused by HC loss. Here, we have conditionally knocked down *Foxg1* specifically in utricular SCs by using Sox9-CreER mice. We found that cKD of *Foxg1* in SCs in the neonatal utricle leads to increased numbers of HCs in both the ES and S regions by inducing SC trans-differentiation. Moreover, cKD of *Foxg1* also induced SC trans-differentiation and thus increased the HC number in an *in vitro* model of neomycin-induced HC damage. The newly generated HCs could survive at least to P30, and the sense of balance was not affected by *Foxg1* cKD. Thus, our results showed that Foxg1 plays similar roles in utricular SCs and HC regeneration as it does in cochlear SCs.

It has been reported that many important genes are expressed in utricular SCs, such as *Sox2*, *Lgr5*, *Sox9*, and *Plp*. In the neonatal mouse utricle, Sox2 is not only expressed in utricular SCs, but also in some utricular type II HCs, while Lgr5 is only expressed in the S region SCs after neomycin treatment [17]. Plp^CreER/+^Rosa26-tdTomato mice were used to lineage trace Plp in the utricle and found that Plp was mainly expressed in SCs in the ES region in the neonatal utricle (Suppementary Figure 2), similar to previous reports [17, 39, 58–60]. Because the purpose of our study was to investigate the effects of Foxg1 in regulating the regeneration of HCs from SCs, the expression patterns of these genes did not meet our requirements. Recent studies have shown that the transcription factor Sox9 is localized in SCs of the late-embryonic inner ear sensory epithelia and in all neonatal SCs [39, 61]. Sox9^CreER/+^Rosa26-tdTomato mice were used in this study to lineage trace Sox9, and we found that Sox9 was expressed in almost all of the SCs in the neonatal mouse utricle, while only 5% of the HCs showed Sox9+ expression. Therefore, we considered Sox9 to be a good marker for utricular SCs.

We observed many more HCs in the Sox9^CreER/+^Foxg1^loxp/loxp^ mouse utricle compared to the Foxg1^loxp/loxp^ control mice (Figure 2A–2C). However, we did not observe any difference in HC number between the Plp^CreER/+^Foxg1^loxp/loxp^ mouse utricle and the Foxg1^loxp/loxp^ control utricle (Figure 2E, 2G). Considering that Plp is only expressed in SCs in the ES region, we speculated that perhaps the regeneration of HCs is mostly from SCs in the S region rather than the ES region. Additionally, we observed increased HC numbers in the S region of the Sox2^CreER/+^Foxg1^loxp/loxp^ mouse utricle compared to the Foxg1^loxp/loxp^ control utricle (Figure 2F, 2H), which supported our hypothesis that it is mainly SCs in the S region that trans-differentiate into HCs.

We also observed that cKD of *Foxg1* in utricular Sox9+ SCs led to increased HCs number in both the S and ES regions of the utricle and that the HC number in *Foxg1* cKD mice utricles remained significantly greater than the control mice at least to P30 (Figure 4A–4C). The adult *Foxg1* cKD mice showed normal vestibular function, including normal swimming behavior and VOR response (Figure 4D–4F), which suggested that the increased number of HCs did not have a negative influence on vestibular function.

HCs can be damaged by many factors, such as aminoglycoside antibiotics, noise exposure, aging, and genetic factors [62]. The inner ear HCs of birds and fish can be regenerated spontaneously after damage, while adult mammalian cochlear HCs in the inner ear cannot be regenerated and utricular HCs have very limited regeneration capacity [17, 63]. Much effort has been put into inducing HC regeneration in the mammalian inner ear, but only limited progress has been achieved [17, 18]. Our *in vivo* data indicate that under normal conditions, Foxg1 plays important roles in the trans-differentiation of SCs in the neonatal mouse utricle. In the *in vitro* neomycin-induced HC damage model, cKD of *Foxg1* in neonatal utricular SCs also increased the HC number through trans-differentiation of SCs, which was consistent with the *in vivo* data. But interestingly, we found that the double positive cells (Myo7a+ and tdTomato+) were only significantly increased in the striolar region of utricle after neomycin injury *in vitro*. We suspected that this might be due to the fact that HCs in the S region is more susceptible to neomycin injury than HCs in the ES region [45, 56], and that SCs in S region are reported to be HC progenitors in utricle which are activated to express Lgr5 and to regenerate HCs after HC damage by neomycin treatment [17]. Our results thus provide new insights into HC regeneration in the adult mammalian utricle after HC damage.

In recent decades, although many important genes have been shown to regulate the proliferation and differentiation of SCs and HC regeneration, the efficiency of HC regeneration in the mammalian cochlea and utricle has remained very limited. It is very possible that many more genes are involved in the process, and their roles are waiting to be discovered. Understanding the detailed mechanisms of HC regeneration and finding ways to increase the efficiency of HC regeneration are fundamental for the development of clinical treatments for hearing loss. Our study shows that *Foxg1* cKD in utricular SCs increases the number of HCs by inducing SCs trans-differentiation under both normal conditions and after neomycin-induced HC injury, which suggests that Foxg1 may be a new candidate gene for regulating HC regeneration in the utricle.

## MATERIALS AND METHODS

### Animals

Sox9-CreER mice were a gift from Prof. Fengchao Wang (National Institute of Biological Sciences, Beijing) [64]. Sox2-CreER mice (Jackson Laboratory, #017593) [65], Plp-CreER mice (Jackson Laboratory, #5975) [17], and Rosa26-tdTomato mice (Jackson Laboratory, #007914) [66, 67] were used in the experiments. The sex of the mice was randomly selected. The Foxg1-floxp mice were a gift from Prof. Chunjie Zhao (Southeast University, Nanjing) [30]. The breeding strategy of mice is shown in Supplementary Figure 1. All procedures about animals were according to protocols that were approved by the Animal Care and Use Committee of Southeast University.

### Surgery-free vestibulo-ocular reflex (VOR) measurement and analysis

P01 mice were intraperitoneally (i.p.) injected with tamoxifen (Sigma, T5648) to activate the Cre enzyme, and surgery-free VOR was measured at P30. Prior to the VOR response measurement, pilocarpine nitrate eye drops (UNIVISION) were added to left eyes of the mice for pupil miosis. The mice were then mounted on the motion platform of the VOR testing system (from Prof Fangyi Chen, Southern University of Science and Technology) [68], and the VOR response was recorded by the cameras as previously described [68]. The VOR measurements were performed at three rotation modes (0.25 Hz 20°, 0.5 Hz 20°, and 1 Hz 20°). In all modes, the rotation stimulus was continuously applied for more than 80 s. To obtain the eye rotation amplitude, we first manually delineated the pupil boundary in the first frame of each video file to generate the matching template. Following this template, the region of interest (contains the pupil) in each frame of the video could be automatically selected. By using the starburst algorithm, we determined the pupil boundary from the region of interest, and then the exact position of the pupil in other frames could be further determined. Subsequently, we applied an ellipse fit to define the center of the pupil. The center position of the pupil reflected the trajectory of the eye movement. The amplitudes were calculated from the recorded eye movement video by using customized software from prof. Fangyi Chen (Southern University of Science and Technology) [68].

### Swimming tests

P01 mice were i.p. injected with tamoxifen to activate the Cre enzyme, and swimming tests were performed at P30. Sox9^CreER/+^Foxg1^loxp/loxp^ /Rosa26-tdTomato mice and the control mice were placed individually in a 1 L beaker filled with water at room temperature, and their swimming behaviors were recorded with a camera. Normal swimming behavior was defined as an elongated, balanced posture with the tail extending caudally, while abnormal swimming behavior was characterized as rolling to one side, excessive circling (tail curling back to the head), floating immobile, and tumbling underwater. We scored the swimming tests according to previous studies as follows [69, 70]: normal swimming, score 0; abnormal swimming, score 1; immobile floating, score 2; underwater tumbling, score 3. We quantified and compared the total swimming score of each group of mice using GraphPad Prism 7 software.

### The PCR of genotyping

All mice were genotyped by using genomic DNA from tail tips of. The tail tips were incubated in 180 μl 50 mM NaOH at 96°C for 1 h for digesting, then 20 μl 1 M Tris-HCl (pH 7.0) was added to the NaOH containing genomic DNA. The genotyping primers were as follows: tdTomato wild type (WT) (F) 5′-AAG GGA GCT GCA GTG GAGT-3′; WT ® 5′-CCG AAA ATC TGT GGG AAGTC-3′; mutant (MUT) (F) 5′-GGC ATT AAA GCA GCG TAT C-3′; MUT ® 5′-CTG TTC CTG TAC GGC ATG G-3′. *Foxg1*: WT (F) 5′-ATA AAG ATTTGC TGA GTT GGA-3′; MUT (F) 5′-GCA TCG CATTGT CTG AGT AGG TG-3′; ® 5′-TGG AGG GGG AGATAG GGC TAT-3′. *Sox9*: WT(F) 5′- CTA GGC CAC AGA ATT GAA AGA TCT-3′; WT ® 5′-GTA GGT GGA AAT TCT AGC ATC ATC C-3′; MUT (F) 5′- GCG GTC TGG CAG TAA AAA CTA TC -3′; MUT ® 5′- GTG AAA CAG CAT TGC TGT CAC TT -3′. *Plp*: WT (F) 5′- CTA GGC CAC AGA ATT GAA AGA TCT-3′; WT ® 5′-GTA GGT GGA AAT TCT AGC ATC ATC C-3′; MUT (F) 5′- AGG TGG ACC TGA TCA TGG AG-3′; MUT ® 5′- ATA CCG GAG ATC ATG CAA GC-3′. *Sox2*: WT (F) 5′-CTA GGC CAC AGA ATT GAA AGATCT-3′; WT ® 5′-GTA GGT GGA AAT TC TAG CAT CA TCC-3′; MUT (F) 5′-GCG GTC TGG CAG TAA AAA CTA TC-3′; MUT ® 5′-GTG AAA CAG CAT TGCTGT CAC TT-3′. The PCR mixes included 3 μl DNA, 0.5 μl primer of each, 10 μl 2× PCR mix (P131-01, Vazyme), and add H_2_O up to 20 μl. The conditions of genotyping PCR as follows: an initial denaturing of 5 min at 95°C, 35 cycles of 30 s denaturation at 95°C, 30 s annealing at 60°C, and 35 s extension at 72°C.

### RNA extraction for PCR

Utricles and cochlea were dissected from the mice to extract total RNA using Trizol Reagent (Life, 15596-018). RNA was reverse transcribed into cDNA (RevertAid First Strand cDNA Synthesis Kit, K1622, Thermo Scientific). For reverse transcription PCR (RT-PCR), The PCR mixes included 1 μl cDNA, 0.5 μl each primer, 10 μl 2× PCR mix (P131-01, Vazyme), and H2O to a total volume of 20 μl. FastStart Universal SYBR Green Master (ROX) kit (Roche, 17747200) were used to perform the real time quantitative PCR (RT-qPCR) to quantify the gene expression levels on a Bio-Rad C1000 Touch thermal cycler. The PCR conditions are as follows: an initial denaturing step of 15 s at 95°C, 35 cycles of 15 s denaturation at 95°C, 60 s annealing at 60°C, and 20 s extension at 72°C. The reference endogenous gene used in this study is *Gapdh.* Gene expression was quantified using the ΔΔCT method. The qPCR primers were as follows: *Gapdh* (F) 5′- AGG TCG GTG TGA ACG GAT TTG -3′; *Gapdh* ® 5′- TGT AGA CCA TGT AGT TGA GGT CA -3′; *Foxg1* (F) 5′- AGC GAC GAC GTG TTC ATC G -3′; *Foxg1* ® 5′- CCC GTT GTA ACT CAA AGT GCT G -3′; *Sox9* (F) 5′- GAG CCG GAT CTG AAG AGG GA -3′; *Sox9* ® 5′- GCT TGA CGT GTG GCT TGT TC -3′.

### Western blot

Utricles and cochlea were dissected to extract total protein with RIPA lysis buffer (FD008, Beyotime) plus protease inhibitor cocktail (04693132001, Roche). The proteins were transferred to polyvinylidene fluoride membranes (ISEQ00010, Millipore) after separation on polyacrylamide gels. The membranes were blocked with 5% skimmed milk. After washing three times with 0.1% Tween in PBS, the membranes were incubated with primary antibodies. The primary antibodies were anti-Foxg1 (Abcam, ab18259, 1:500 dilution), anti-Sox9 (Santa Cruz, sc-166505, 1:1000 dilution), and anti-Gapdh (KC-5G4, Kangchen, 1:2000 dilution). Gapdh was used as the reference protein. After washing again three times with 0.1% Tween in PBS, the membranes were incubated with peroxidase-conjugated goat anti-rabbit and goat anti-mouse secondary antibodies. The target proteins were detected with a SuperSignal West Dura chemiluminescent substrate kit (Thermo Scientific, 34075) and visualized on a Tanon-5200 imaging system.

### Immunostaining and image acquisition

The utricle was dissected (WPI forceps) in cold HBSS and fixed in 4% paraformaldehyde for 1 h at room temperature (RT). The utricle was washed three times with PBS and then blocked with the medium (5% donkey serum, 0.5% Triton X100, and 1% bovine serum albumin in pH 7.4 PBS) for 1 h at RT, then incubated with primary antibodies diluted in the medium (5% donkey serum, 0.1% Triton X100, and 1% bovine serum albumin in pH 7.4 PBS) at 4°C overnight. The utricle was then washed three times with 0.1% Triton X100 in pH 7.4 PBS and incubated with secondary antibody (Invitrogen) or phalloidin (A12379, Invitrogen), 1:400 diluted in the medium (1% bovine serum albumin and 0.1% Triton X100 in pH 7.4 PBS) for 1 h at RT. After washed 3 times, the utricle was mounted with antifade fluorescence mounting medium (DAKO, S3023). The primary antibodies were as follows: anti-Myo7a (DSHB, #138-1; myo7a; Proteus Bioscience, #25-6790; both 1:1000), anti-Sox9 (Santa Cruz, sc-166505, 1:200), anti-Sox2 (Santa Cruz, sc-17320, 1:400). All samples were scanned by Zeiss microscope (LSM 710, Zeiss, Heidenheim, Germany) for image acquisition.

### *In vivo* cKD of *Foxg1* in Sox9+ cells, Plp+, and Sox2+ cells in the mouse utricle

Sox9^CreER/+^Foxg1^loxp/loxp^ mice, Plp^CreER/+^Foxg1^loxp/loxp^ mice, and Sox2^CreER/+^Foxg1^loxp/loxp^ mice were bred to specifically knock down *Foxg1* in Sox9+ cells, Sox2+ cells, and Plp+ cells, respectively. In order to activate the Cre enzyme, P01 mice were injected with tamoxifen. All procedures above were performed as described previously [38]. Same dose of tamoxifen were injected to the mice in control group. The utricles were examined from mice that were sacrificed at different ages.

### *In vivo* lineage tracing of Sox9+ SCs in the utricle

Sox9^CreER/+^Foxg1^loxp/loxp^ mice were crossed with Foxg1^loxp/loxp^Rosa26-tdTomato mice to obtain Sox9^CreER/+^Foxg1^loxp/loxp^Rosa26-tdTomato mice. These triple positive mice were used to lineage trace Sox9+ SCs of utricle. Sox9^CreER/+^Foxg1^loxp/loxp^Rosa26-tdTomato mice were i.p. injected with tamoxifen at P01 to activate the Cre enzyme, and then sacrificed at P08. Same dose of tamoxifen were injected to Sox9^CreER/+^Rosa26-tdTomato mice that used as controls.

### Data quantification

To quantify the data, such as HC number, SC number, and double or triple-positive cell number, we randomly measured two 63× magnification images of the utricle in the S region and ES region as representative images. The utricle was always in the center of the image (0.01 mm^2^ utricle area per image). We counted the total numbers of HCs, SCs, or multiply positive cells in the image, averaged the numbers of two images for the S region and ES region respectively, and presented the figure as numbers per 0.01 mm^2^. All the experiments in this study, analysts are blind to the treatment conditions.

### Statistical analysis

Three independent experiments were performed in each experimental condition. “N” in the figures refers to the experimental repetitions of real-time qPCR or the number of mice as shown in the legends of figure. We used two-tailed, unpaired Student’s t-tests to determine the significance statistic. We used GraphPad Prism 7 software to analyze the data, and all data were shown as means ± standard errors of the means (SEM). Statistically significant was considered when value of P < 0.05.

## Supplementary Material

Supplementary Figures

Supplementary Video 1
