# Supplementary material for: Knockdown of Foxg1 in Sox9+ supporting cells increases the trans-differentiation of supporting cells into hair cells in the neonatal mouse utricle
Source: Aging (Albany NY). 2020 Oct 24;12(20):19834–51. doi: 10.18632/aging.104009 (PMC7655167; doi:10.18632/aging.104009)
Supplement: Supplementary Figures [file aging-12-104009-s001..pdf]

SUPPLEMENTARY FIGURES

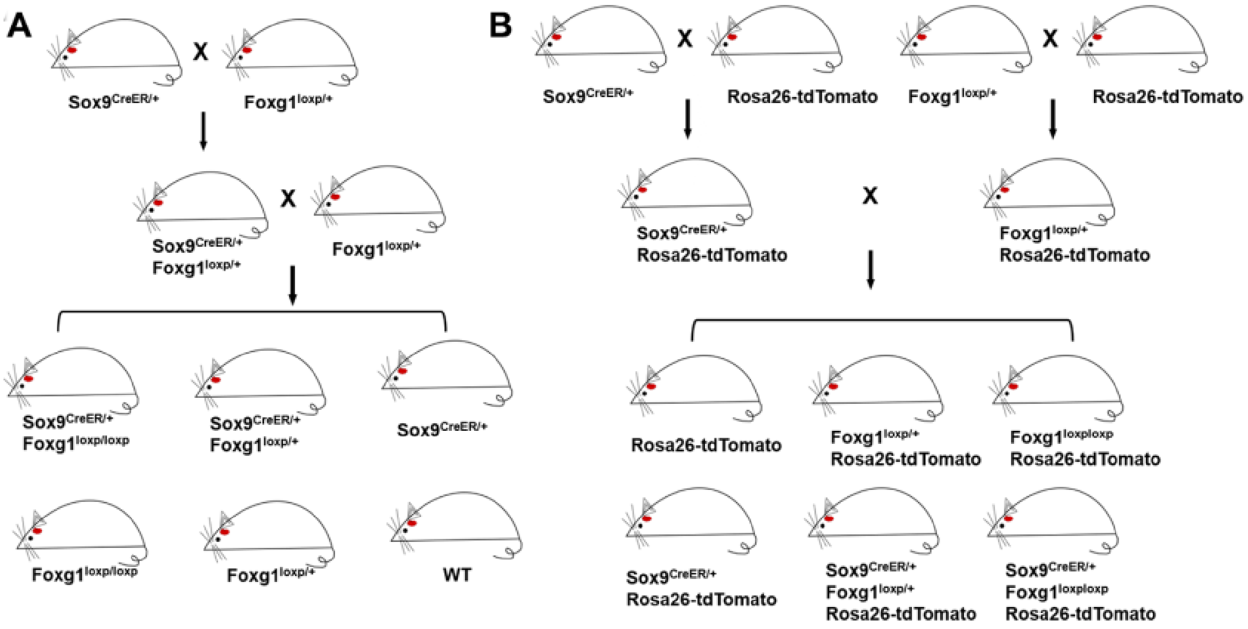

Supplementary Figure 1. The mouse breeding strategy. (**A**) Breeding strategy for Sox9$^{CreER/+}$Foxg1$^{loxp/loxp}$ mice. (**B**) Breeding strategy for Sox9$^{CreER/+}$Foxg1$^{loxp/loxp}$Rosa26-tdTomato mice.

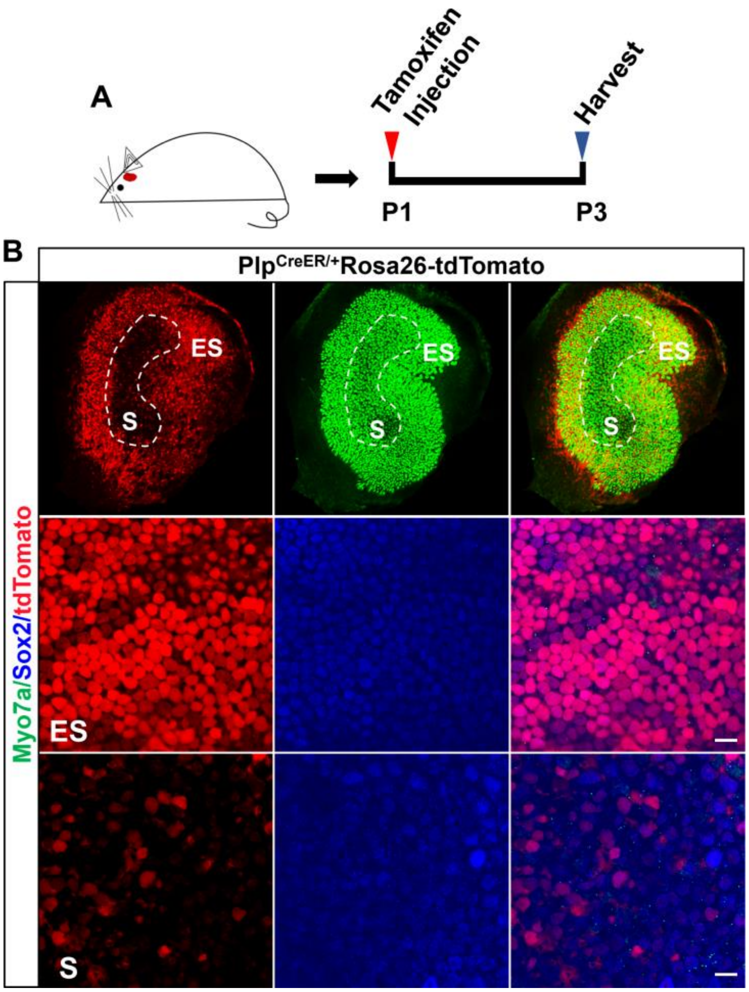

Supplementary Figure 2. Expression of Plp and Sox2 in the neonatal mouse utricle. (**A**) Tamoxifen was i.p. injected into P1 Plp9$^{CreER/+}$Rosa26-tdTomato mice to activate the Cre enzyme, and the mice were sacrificed at P3. (**B**) Lineage tracing images of Plp+ SCs (red) in Plp9$^{CreER/+}$Rosa26-tdTomato mice. Myo7a (green) and Sox2 (blue) were used as HC and SC markers, respectively. For all experiments, the scale bar at high magnification is 10 μm.
